# Genetic and Phenotypic Characterization of a Rabies Virus Strain Isolated from a Dog in Tokyo, Japan in the 1940s

**DOI:** 10.3390/v12090914

**Published:** 2020-08-20

**Authors:** Tatsuki Takahashi, Maho Inukai, Michihito Sasaki, Madlin Potratz, Supasiri Jarusombuti, Yuji Fujii, Shoko Nishiyama, Stefan Finke, Kentaro Yamada, Hiroki Sakai, Hirofumi Sawa, Akira Nishizono, Makoto Sugiyama, Naoto Ito

**Affiliations:** 1 The United Graduate School of Veterinary Sciences, Gifu University, Gifu 501-1193, Japan; w5110009@edu.gifu-u.ac.jp (T.T.); shiroki@gifu-u.ac.jp (H.S.); sugiyama@gifu-u.ac.jp (M.S.); 2 Laboratory of Zoonotic Disease, Faculty of Applied Biological Sciences, Gifu University, Gifu 501-1193, Japan; u8025006@edu.gifu-u.ac.jp (M.I.); shnishiy@gifu-u.ac.jp (S.N.); 3 Division of Molecular Pathobiology, Research Center for Zoonosis Control, Hokkaido University, Sapporo 001-0020, Japan; m-sasaki@czc.hokudai.ac.jp (M.S.); h-sawa@czc.hokudai.ac.jp (H.S.); 4 Institute of Molecular Virology and Cell Biology, Federal Research Institute for Animal Health, Friedrich-Loeffler-Institut, 17493 Greifswald, Germany; madlin.potratz@fli.de (M.P.); stefan.finke@fli.de (S.F.); 5 Graduate School of Bioagricultural Science, Nagoya University, Nagoya 464-8601, Japan; yb520286@edu.gifu-u.ac.jp; 6 Joint Graduate School of Veterinary Sciences, Gifu University, Gifu 501-1193, Japan; y5201102@edu.gifu-u.ac.jp; 7 Department of Microbiology, Faculty of Medicine, Oita University, Oita 879-5593, Japan; kentaro-y@cc.miyazaki-u.ac.jp (K.Y.); a24zono@oita-u.ac.jp (A.N.); 8 Laboratory of Veterinary Pathology, Faculty of Applied Biological Sciences, Gifu University, Gifu 501-1193, Japan; 9 Gifu Center for Highly Advanced Integration of Nanosciences and Life Sciences, Gifu University, Gifu 501-1193, Japan

**Keywords:** rabies virus, fixed virus, street virus, Komatsugawa, Arctic-related clade, pathogenesis

## Abstract

The rabies virus strain Komatsugawa (Koma), which was isolated from a dog in Tokyo in the 1940s before eradication of rabies in Japan in 1957, is known as the only existent Japanese field strain (street strain). Although this strain potentially provides a useful model to study rabies pathogenesis, little is known about its genetic and phenotypic properties. Notably, this strain underwent serial passages in rodents after isolation, indicating the possibility that it may have lost biological characteristics as a street strain. In this study, to evaluate the utility of the Koma strain for studying rabies pathogenesis, we examined the genetic properties and in vitro and in vivo phenotypes. Genome-wide genetic analyses showed that, consistent with previous findings from partial sequence analyses, the Koma strain is closely related to a Russian street strain within the Arctic-related phylogenetic clade. Phenotypic examinations in vitro revealed that the Koma strain and the representative street strains are less neurotropic than the laboratory strains. Examination by using a mouse model demonstrated that the Koma strain and the street strains are more neuroinvasive than the laboratory strains. These findings indicate that the Koma strain retains phenotypes similar to those of street strains, and is therefore useful for studying rabies pathogenesis.

## 1. Introduction

Rabies is a viral zoonosis that affects all mammal species including humans and is caused by infection with highly neurotropic lyssaviruses including rabies virus (RABV). This disease is characterized by a long and inconstant incubation period (usually 20 to 90 days in humans) [1], severe neurological signs and a high case fatality rate, almost 100%. It is distributed worldwide with only a limited number of rabies-free countries including the United Kingdom, New Zealand, Australia and Japan [2,3]. Although rabies is preventable by vaccination, it kills approximately 59,000 people every year, mostly in developing countries in Asia and Africa [3], mainly due to the absence of an effective therapy, and also the insufficient dissemination of a current post-exposure prophylaxis, which requires rabies vaccination up to five times in one month [4]. Thus, there is an urgent need for the development of both a therapeutic approach and a novel prophylaxis method that would, for example, reduce the dependence on vaccination. To achieve these goals, it is important to understand the pathogenesis of rabies, namely, the mechanisms of RABV infection at the molecular level.

RABV is classified into the genus *Lyssavirus* of the family *Rhabdoviridae* within the order *Mononegavirales*. The genome of RABV is a non-segmented negative-sense RNA of about 12 kb, encoding five viral proteins: nucleoprotein (N protein), phosphoprotein (P protein), matrix (M) protein, glycoprotein (G protein), and large (L) protein [5]. The N protein binds to genomic RNA to form a ribonucleoprotein (RNP) structure, which functions as a template for viral RNA synthesis by the polymerase L protein and its cofactor P protein. The M protein physically interacts with the RNP complex and participates in budding of the progeny virion. The G protein forms spikes that project from the lipid-bilayer envelope, which enwraps the RNP-M protein complex, and is involved in receptor binding on and internalization into host cells. Notably, the N, P and M proteins also play important roles in evasion of host innate immunity [6,7,8,9,10,11]. Considering their essential roles in viral replication and immune evasion, it appears reasonable to assume that all of these viral proteins contribute to rabies pathogenesis.

The pathogenesis of rabies exclusively depends on the spread of RABV infection to and in the nervous system of the host individual. In general, RABV excreted into saliva of a rabid animal is transmitted via a bite wound [1]. After penetration into the body, the virus infects peripheral nerves and then spreads to and in the central nervous system (CNS), resulting in severe neurological signs and lethal outcome. To date, molecular mechanisms of rabies pathogenesis have been studied mainly by using vaccine and laboratory strains (fixed strains) including CVS, ERA, SAD and Nishigahara. Such studies using fixed strains have demonstrated that mutations at specific positions in the P and G proteins affect viral neuroinvasiveness [12,13,14], which is defined as an ability of the virus to spread from a peripheral site to the CNS, and also that certain mutations in the N, P, M and G proteins alter viral neurovirulence [15,16,17,18,19,20,21], the ability of the virus to spread in the CNS and to cause lethal disease.

All of these findings indicate the importance of the respective viral proteins in the pathogenesis at the molecular level. However, the findings may not always reflect the pathogenic mechanism of natural rabies infection because the fixed strains have been established by serial passages of a field RABV strain (street strain) in animal brains and, in many cases, cultured cells, potentially causing great changes in the genetic properties and thereby in the biological phenotypes. In fact, it is accepted that fixed strains generally have a higher level of neurotropism, the ability of the virus to propagate in neurons or neuronal cell lines, and a lower level of neuroinvasiveness than do street strains [22,23]. In addition, a very recent study on cell tropism of several RABV strains in the mouse brain strongly suggested that immune evasion mechanisms in the brain differ between fixed and street strains [24]. The results of these studies highlight the importance of molecular studies with street strains for elucidation of the pathogenesis of natural rabies infection and thereby for development of therapeutic and prophylactic approaches for rabies.

In the field, street RABVs with different genetic backgrounds evolve distinctively while retaining the intrinsic mechanisms for the pathogenesis, indicating that each virus strain has biological phenotypes, some of which are unique to the strain and others of which are conserved among all street RABVs in nature. Therefore, to obtain clues for the development of a therapeutic approach that is universally effective in all rabies cases, it is necessary to exhaustively characterize multiple street strains with different genetic origins, and then to pinpoint a key phenomenon that commonly contributes to their pathogenesis. However, very little is known about the pathogenic mechanisms of street RABV infection. In addition, there is a bias on the virus strains that have been genetically and phenotypically characterized so far: although genetic and biological properties of several street strains including RABV-Dog, RABV-Fox, 1088, 8743THA, GD-SH-01, QS-05 and SHBRV-18 have been studied [11,23,24,25,26,27,28,29,30], all of these strains belong to the clade Cosmopolitan, Asian or Bats, only three of the eight major clades that have been reported [31]. This indicates the necessity for exhaustive studies on street strains that are classified into the other clades including the Africa-2 and -3, Indian subcontinent, and Arctic-related clades. 

The RABV strain Komatsugawa (abbreviated as Koma here) was originally isolated from a dog in Tokyo in the 1940s by Dr. Hiroshi Sazawa, the National Institute of Animal Health [32,33,34], before eradication of rabies in Japan in 1957. Since then, this strain has contributed to various rabies studies in Japan as the only existent Japanese street strain [32,33,35,36,37]. Phylogenetic analyses based on the N gene nucleotide sequences demonstrated that the Koma strain is classified into the Arctic-related clade, and is genetically closely related to virus strains isolated from animals and a human in Primorsky Krai, Russia [33,34,38]. This indicates the possibility that genetic and phenotypic characterization of the Koma strain would contribute to an understanding of the intrinsic mechanisms for rabies pathogenesis.

Meanwhile, the genetic and phenotypic properties of the Koma strain remain to be fully elucidated. More specifically, the nucleotide sequence of the complete genome, except for the N gene region, has not yet been determined. In addition, the phenotypic properties of this strain, including its pathogenicity in mice, have not been systematically studied. Notably, after the original isolation from a dog, the Koma strain has been maintained through more than 20 intracerebral passages in mice after a total of 19 brain-to-periphery (hindlimb) passages in guinea pigs [32], raising the concern that the passage process may have degraded biological characteristics of the strain as a street strain. In fact, Arai et al. [33,35] previously categorized this strain as a fixed strain based on this history of passages in rodents. We therefore believe that biological characterization of the Koma strain would provide clues to understand the natural history of this strain, as well as the phenotypic differences between fixed and street strains.

In this study, to obtain insights into the phylogenetic origin of the Koma strain and also to evaluate the utility of this strain for studying rabies pathogenesis, we comprehensively examined genetic and phenotypic properties of this RABV strain. Namely, we investigated the genetic features of the Koma strain and its phylogenetic relationships to other RABV strains after determining its complete genome sequence by next-generation sequencing. Furthermore, we examined by both in vitro and in vivo experiments its biological phenotypes, including its pathogenicity in mice, and compared its biological phenotypes with those of representative street and fixed strains.

## 2. Materials and Methods 

### 2.1. Cells and Virus Strains

Mouse neuroblastoma C1300 (NA) cells [39] were maintained in Eagle’s minimal essential medium (E-MEM) (Wako, Osaka, Japan), supplemented with 10% fetal calf serum. 

The RABV strain Koma was originally isolated from a rabid dog in Tokyo, Japan in the 1940s and has been maintained by intracerebral (i.c.) passages in mice after a total of 19 brain-to-periphery passages in guinea pigs [32]. The Koma strain used in this study was cloned by limited dilution two times in NA cells from an infected mouse brain at the 24th passage. The street strains 1088 and RABV-Dog, which were originally isolated from a woodchuck in the United States and a dog in Azerbaijan, respectively, were rescued from infectious cDNA [25,26], as previously reported [26,40]. The fixed strains CVS, Nishigahara, and ERA were also used in this study. Virus stocks of all of the RABV strains were propagated in NA cells.

### 2.2. Complete Genome Sequencing of Koma Strain

Viral genome RNA was extracted from a virus stock of the Koma strain by using a TRI REAGENT LS (Molecular Research Center, Inc., Cincinnati, OH, USA) and was subject to reverse transcription (RT) using SuperScript III reverse transcriptase (Invitrogen, Carlsbad, CA, USA) and random hexamer (Invitrogen) following the manufacturer’s instructions. Using the RT product as a template, three cDNA fragments, which collectively covered almost the entire region of the genome, were amplified by PCR using KOD One PCR Master Mix (TOYOBO, Osaka, Japan) and three sets of primers (Table S1). The PCR amplifications were performed for 35 cycles of denaturation at 98 °C for 10 s, annealing at 62 °C for 5 s, and extension at 68 °C for 25 s. These cDNA fragments were pooled and sheared using a Covaris S2 focused-ultrasonicator (Covaris, Woburn, MA, USA). A 200-base-read library was prepared with the Ion Plus Fragment Library kit (Ion Torrent; Thermo Fisher Scientific, Waltham, MA, USA) and sequenced using the Ion PI Hi-Q Sequencing 200 kit, the Ion PI Chip Kit v3 and the Ion Proton sequencer (Ion Torrent) according to the manufacturer’s protocol. The sequence reads were assembled by using the CLC Genomics Workbench version 10.0 software (CLC bio, Aarhus, Denmark). As a reference sequence for the sequence assembly, the genome sequence of RV303 strain (GenBank accession number: KY860613) was used.

To determine both the 3′ and 5′ terminal sequences of the Koma genome, the extracted genome RNA mentioned above was circularized by using T4 RNA ligase (NEB, Ipswich, MA, USA), in accordance with the manufacturer’s recommendations and then a cDNA fragment containing both terminal regions was amplified by nested RT-PCR. The first and second PCRs were conducted by using the PrimeScript One Step RT-PCR kit (Takara, Shiga, Japan) and KOD One PCR Master Mix (TOYOBO), respectively. Sequences of the primers used for this RT-PCR are shown in Table S1. The one step RT-PCR was carried out at 50 °C for 30 min and 94 °C for 2 min, followed by 30 cycles of 94 °C for 30 s, 58 °C for 30 s, and 72 °C for 1 min. The second PCR amplification was performed for 30 cycles of 98 °C for 10 s, 55 °C for 5 s, and 68 °C for 5 s. Sequences of the primers used for this RT-PCR are shown in Table S1. The nucleotide sequence of the cDNA fragment was determined by BigDye-Terminator ver. 3.1 and ABI3130 genetic Analyzer (Applied Biosystems, Waltham, MA, USA).

The complete genome sequence of the Koma strain determined in this study was deposited in the GenBank database with accession number LC553558.

### 2.3. Genetic and Phylogenetic Analyses

The genome sequences of the Koma strain and a total of 63 reference RABV strains, which were obtained from GenBank (Table S2), were aligned by using the MUltiple Sequence Comparison by Log-Expectation (MUSCLE) software (Drive5, Mill Valley, CA, USA). For phylogenetic analysis, the maximum-likelihood method with the GTR+G+I model was applied by using Molecular Evolutionary Genetics Analysis (MEGA) software ver. 10.1 [41] (Pennsylvania State University, State College, PA, USA). The MEGA software was also used to determine percent homology between nucleotide or amino acid sequences of the Koma and RV303 strains.

### 2.4. Growth of Each Virus Strain in NA Cells

NA cells grown in a 6-well tissue culture plate were inoculated with 0.5 mL/well of each virus strain at a multiplicity of infection (MOI) of 0.001. After adsorption of virus for 60 min, the cells were washed three times with 3 mL/well of Hanks’ balanced salt solution (Nissui, Tokyo, Japan). Afterward, the cultures were replenished with 6 mL/well of fresh medium and incubated 37 °C. The culture media were collected at 0, 1, 3, 5 days post-infection (dpi), and then infectious viruses in the culture supernatant were titrated by focus assays on NA cells as previously reported [12]. The viral titer was calculated as focus-forming units [FFU]/mL. This assay was carried out in triplicate.

### 2.5. Focus Formation by Each Virus Strain in NA Cells

NA cells grown in a 24-well plate were inoculated with each virus strain at an MOI of 0.0002 and then overlaid with E-MEM containing 1% methylcellulose (NACALAI TESQUE, Kyoto, Japan). At 3 days post-inoculation, the infected cells were fixed with 4% paraformaldehyde (Wako) for 60 min and 100% methanol (Wako) for 1 min and then immunostained by using an anti-RABV N protein monoclonal antibody as previously reported [42]. Virus foci on the immunostained samples were photographed by using the Biozero fluorescence microscope BZ-8000 series (Keyence, Osaka, Japan). A total of 50 foci on the digital images, which had randomly been chosen, were analyzed by Image J software ver. 1.52 (National Institutes of Health, Bethesda, MD, USA) to determine the average focus area.

### 2.6. Pathogenicity of Each Virus Strain In Vivo

Six-week-old male ddY mice (Japan SLC, Inc., Shizuoka, Japan) were used for all in vivo experiments. To determine lethal dose 50 (LD_50_), mice were inoculated via the i.c. or intramuscular (i.m.) route (into the left thigh muscle) with 0.03 mL or 0.1 mL, respectively, of serial 10-fold dilutions of each virus strain. Mice inoculated with the Koma strain and street strains 1088 and RABV-Dog were observed daily for 50 days, while mice inoculated with fixed strains (Nishigahara, CVS, and ERA) were observed for 21 days. Mice were euthanized when they showed a lack of righting reflex (mice unable to right themselves within 10 s after being placed on their side). The LD_50_ of each virus strain was calculated on the basis of the method of Reed and Muench [43]. All animal experiments in this study were conducted in accordance with the Regulations for Animal Experiments in Gifu University; the protocols were approved by the Committee for Animal Research and Welfare of Gifu University (approval no. H30-201 and H30-202) on 7 March 2019.

### 2.7. Examination of Incubation Period in Mice

A total of 10 mice per group were inoculated intramuscularly with 0.1 mL of 3.0 LD_50_ (by i.m. route) of each virus strain and were observed daily for 50 days. We defined the incubation period as the period from the day of virus inoculation to the day of onset of disease, when the body weight of an inoculated mouse decreased by 5% or more, compared to the weight on the previous day.

### 2.8. Immunohistochemical Analysis

The brains of mice infected with 3.0 LD_50_ of each virus strain via the i.m. route were collected on the day of euthanasia and fixed in 4% paraformaldehyde before being embedded in paraffin. Deparaffinized sections were pretreated with 0.1% trypsin (Sigma-Aldrich) and then blocked with 2% normal donkey serum (Wako). The sections were incubated with an anti-P protein rabbit polyclonal antibody reported previously [42] and with Alexa Fluor 488-conjugated anti-rabbit IgG (Invitrogen). Nuclei were stained with Mounting Medium with DAPI (Vector, Burlingame, CA, USA). The brain tissues were photographed by using the EVOS M7000 Imaging System (Thermo Fisher Scientific).

### 2.9. Statistical Analysis

One-way or two-way analysis of variance (ANOVA) with Dunnett’s multiple-comparison test was conducted to determine statistical significance by using GraphPad prism ver. 8.3 (GraphPad Software, San Diego, CA, USA). *p* Values of < 0.05 were considered statistically significant.

## 3. Results

### 3.1. Genome Organization of Koma Strain

**Figure 1:**
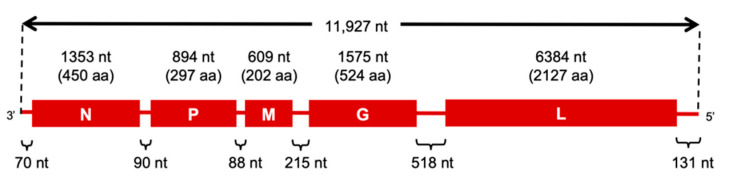
Schematic diagram of the genome organization of the Koma strain. Boxes and lines show open reading frames and non-coding regions, respectively. nt: nucleotides, aa: amino acids.

First, we determined the complete genome sequence of the Koma strain and examined its genome organization. We found that the genome was composed of 11,927 nucleotides (nt), including open reading frames of the N, P, M, G and L genes, which consist of 1353 nt, 894 nt, 609 nt, 1575 nt and 6384 nt, respectively (Figure 1). Thus, it was estimated that N, P, M, G and L proteins of the Koma strain were composed of 450 amino acids (aa), 297 aa, 202 aa, 524 aa and 2127 aa, respectively. The respective genes were flanked by transcription start and stop signals (AACAYYHCU and WGAAAAAAA, respectively, indicated as positive-sense RNA, Y, W and H representing C/U, A/U and A/C/U, respectively). These findings indicate that the Koma strain has the same genome organization as that of other RABV strains.

### 3.2. Phylogenetic Relationships between Koma Strain and Other RABV Strains

**Figure 2:**
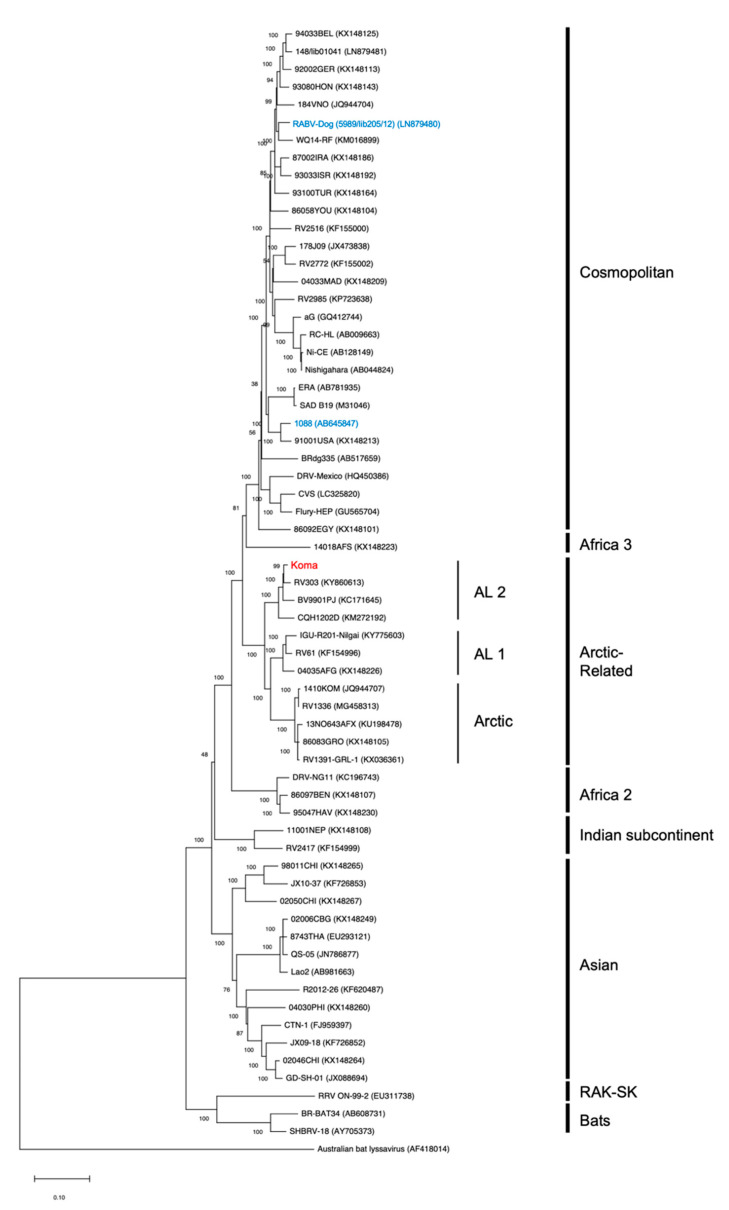
Phylogenetic relationships of the Koma strain with other rabies virus (RABV) strains. Complete genome nucleotide sequences of a total of 63 strains, which are representative strains of the major phylogenetic clades previously reported [31], were obtained from the GenBank database and were subject to phylogenetic analysis by the maximum-likelihood method, together with the genome sequence of the Koma strain. The genetic analysis software Molecular Evolutionary Genetics Analysis (MEGA) version 10.1 was used for phylogenetic analysis. Numbers at nodes and in parentheses represent bootstrap values obtained from 1000 replicates and the accession numbers in the GenBank database. The bar indicates 0.10 substitutions per nucleotide position. The Koma strain and two street strains (1088 and RABV-Dog) are shown in red and blue, respectively.

Previously, phylogenetic analyses based on partial or complete nucleotide sequences of the N gene demonstrated that the Koma strain is genetically associated with Arctic-related RABVs [33,34,38]. However, a genome-wide phylogenetic analysis has not been conducted for the Koma strain, due to the lack of information on its complete genome sequence. In this study, by using the complete genome nucleotide sequences, we examined the phylogenetic relationships of Koma strain with representative RABV strains from eight phylogenetic clades (Cosmopolitan, Asian, Arctic-related, Africa 2, Africa 3, Indian subcontinent, RAK-SK and Bats) [31]. The resulting phylogenetic tree indicated that the Koma strain was classified into the Arctic-like 2 (AL2) subclade of the Arctic-related clade, together with three street strains, CQH1202D, BV9901PJ, and RV303 previously isolated in China, Korea and Russia, respectively (Figure 2). Of these three strains, The RV303 strain (GenBank accession number: KY860613.1), which was isolated from a raccoon dog in Primorsky Krai, Russia in 1989, was the most closely related to the Koma strain. These findings indicate that, consistent with results of previous phylogenetic analyses based on the N gene sequences, Koma strain is closely related to RABVs that are classified into the Arctic-related clade. 

### 3.3. Genetic Properties of Koma Strain

Next, we comprehensively characterized the genome of the Koma strain by comparing its genome with that of the street strain RV303, which showed the closest phylogenetic relationship with the Koma strain (Figure 2). We found that the genome of the Koma strain was 1-nt longer than that of RV303 strain (11,926 nt), due to a nucleotide insertion in the 5’ terminal non-coding region (at nucleotide position 11,915). Their complete genome nucleotide sequences were 98.2% identical (Table 1). The respective viral genes shared homologies of 97.2% to 98.5% and 98.0% to 99.8% at the nucleotide and amino acid levels, respectively. While the amino acid sequence of the P protein showed the lowest homology (98.0%), those of all other proteins showed homology of 99.0% or higher.

**Figure 3:**
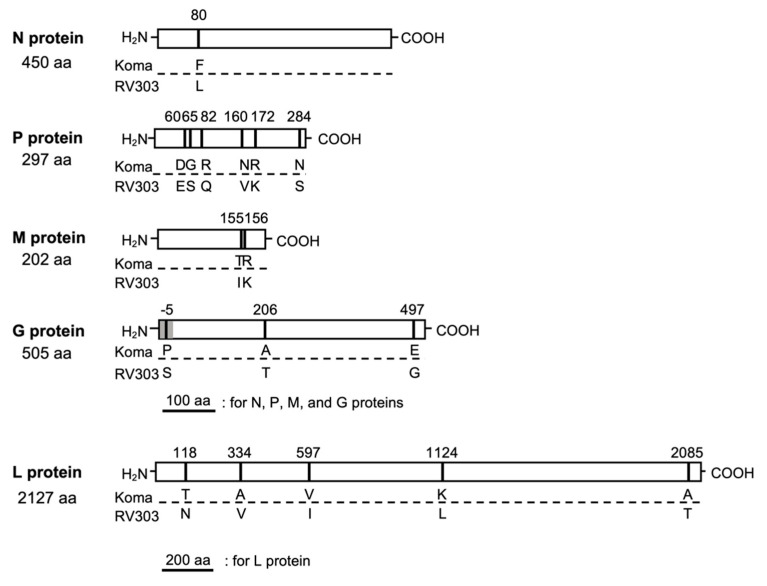
Amino acid substitutions in N, P, M, G, and L proteins between Koma and RV303. Vertical bars with numbers on each protein indicate positions of the substitutions. Amino acid residues of the Koma strain (upper) and RV303 strain (lower) at the substitution sites are shown as a single letter code. The amino acid number in the G protein is assigned to the mature form that does not contain an N-terminal signal peptide of 19 aa (shaded).

A comparison of the amino acid sequences of all viral proteins revealed a total of 17 amino acid differences between the Koma and RV303 strains (Figure 3). In the N protein, an amino acid difference was found only at position 80. In the P protein, there were six differences, three of which were located in the amino-terminal region at positions 60 to 82, and the remaining three were in the carboxy-terminal half. In the M protein, two amino acid differences were found at positions 155 and 156. The G protein had a total of three differences at positions −5, 206, and 497, which were located within the signal peptide region (amino acid positions −19 to −1), ectodomain (1 to 439), and cytoplasmic tail (462 to 504) [5], respectively. In the L protein, there were five differences, three of which were in the amino-terminal half. Notably, amino acid residues that are important for viral pathogenicity of several fixed strains, including Arg at position 333 in the G protein [15], Phe and Tyr at positions 273 and 394 in the N protein [8,19] and others in the P, M and G proteins [44,45,46], were perfectly conserved between the Koma and RV303 strains.

Taken together, the findings described above indicate that there are only minor differences between genetic properties of the Koma strain and the street strain RV303.

### 3.4. Number and Position of Potential N-Glycosylation Sites on the Koma G Protein

**Figure 4:**
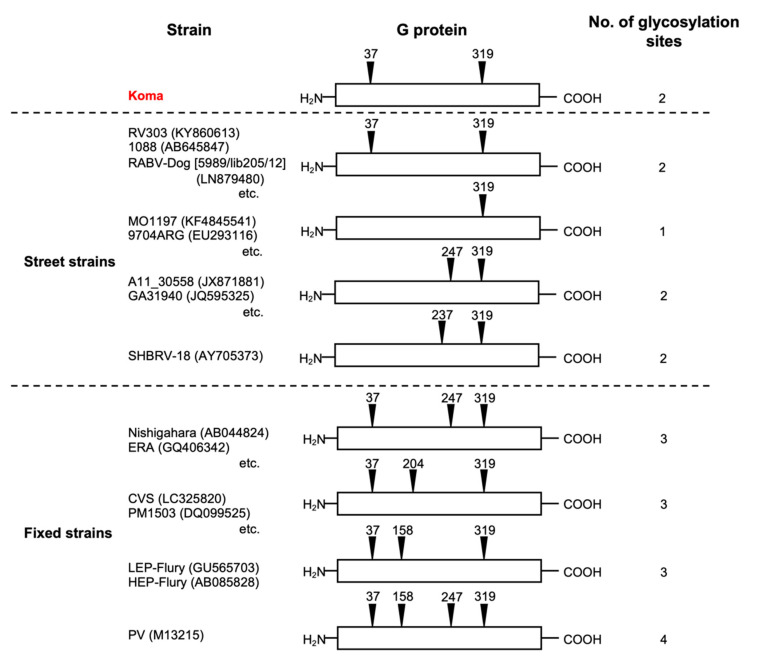
Positions and total numbers of potential N-glycosylation sites in the G protein of RABV strains. Black triangles with numbers represent the potential N-glycosylation sites. Information on the glycosylation sites in the street and fixed strain G proteins was obtained from articles by Yamada et al. [23] and Hamamoto et al. [47].

It was previously reported that street strains generally have two (or one in some cases) potential sites for N-glycosylation (Asn-X-Ser/Thr, X being any amino acid except Pro) on their G protein, while fixed strains have three (or four in a few cases) potential sites on the G protein [23,47], suggesting that the number of glycosylation sites is a key factor that determines the difference between biological properties of street and fixed strains. Based on the amino acid sequence of the Koma G protein, we examined the number and position of potential N-glycosylation sites on the protein and found that, as in the case of the majority of street strains, the Koma strain has two potential sites at positions 37 and 319 on the G protein (Figure 4). This finding indicates the possibility that the Koma strain has biological properties similar to those of street strains, rather than those of fixed strains.

### 3.5. Growth of Koma Strain in Neuroblastoma NA Cells

To check whether the Koma strain indeed has biological properties similar to those of the street strains, we compared the in vitro and in vivo phenotypes of the Koma strain with the phenotypes of two street strains (1088 and RABV-Dog) and with those of three fixed strains (Nishigahara, CVS and ERA). 

**Figure 5:**
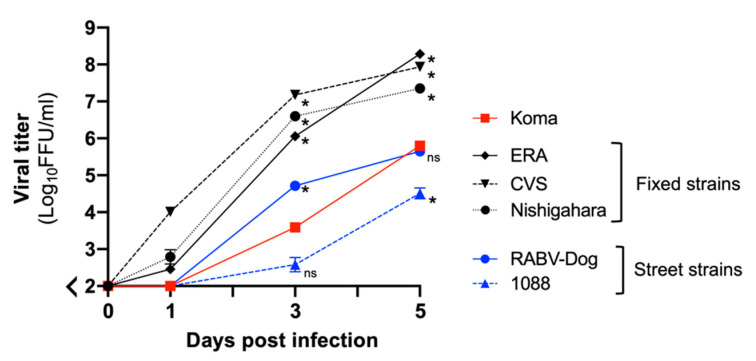
Growth curves for the Koma strain and representative street and fixed strains in mouse neuroblastoma C1300 (NA) cells. NA cells were inoculated with each of the virus strains at an MOI of 0.001. Viruses in the culture supernatants were collected at 0, 1, 3 and 5 dpi and titrated in NA cells by a focus assay. This assay was performed in triplicate, and the values in the graph are shown as means ± standard errors of the means. *, Significant differences vs. the Koma strain at a *p* value of < 0.05; ns, not significant (*p* ≥ 0.05)**.**

First, we examined and compared the abilities of these strains to grow in mouse neuroblastoma NA cells (Figure 5). We found that all of the virus strains grew exponentially in the cells, but their highest titers clearly differed between the street and fixed strains: titers of the fixed strains reached over 1.0 × 10^7^ FFU/mL at 5 dpi, whereas titers of the two street strains were less than 1.0 × 10^6^ FFU/mL. Importantly, the titer of the Koma strain at 5 dpi was 6.7 × 10^5^ FFU/mL, which was significantly lower than the titers of all of the fixed strains (*p* < 0.05) and was comparable to the titer of the street strain RABV-Dog (*p* ≥ 0.05). These data indicate that the growth property of the Koma strain in NA cells is more similar to the properties of the street strains than those of the fixed strains. 

### 3.6. Cell-to-Cell Spread of Koma Strain in NA Cells

**Figure 6:**
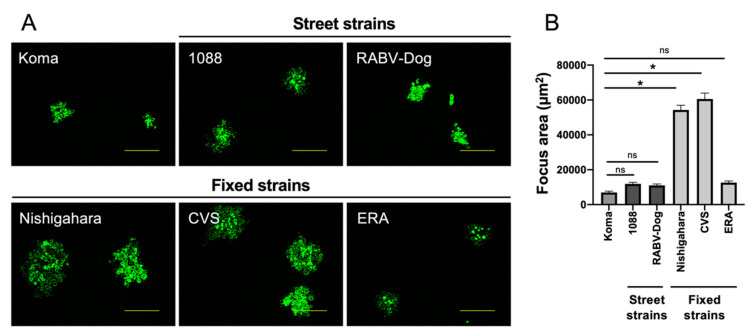
Focus formation by Koma and representative street and fixed strains in mouse neuroblastoma C1300 (NA) cells. (**A**) At 3 days after inoculation of NA cells with each virus strain at an MOI of 0.0002, the cells were fixed and immunostained with an anti-RABV N protein antibody. The scale bars correspond to 200 µm. (**B**) Fifty foci of each strain were randomly selected to quantify their areas by Image J software. Each column represents the average area (± standard errors of the means). *, Significant differences vs. the Koma strain at a *p* value of < 0.05; ns, not significant (*p* ≥ 0.05)**.**

Next, we compared the efficiencies of cell-to-cell spread of the virus strains in NA cells by checking their focus sizes in the cells (Figure 6). We observed that the focus size in NA cells inoculated with the fixed strains varied depending on the strain: the Nishigahara and CVS strains formed larger foci than did ERA strain (Figure 6A). On the other hand, the street strains 1088 and RABV-Dog both formed smaller foci than those formed by the Nishigahara and CVS strains. Importantly, the Koma strain also formed analogous foci in size to these street strains, thus forming smaller foci than did the fixed Nishigahara and CVS strains. Quantification of the focus area of the respective strains supported the above observations (Figure 6B). These findings indicate that the cell-to-cell spread efficiency of the Koma strain is comparable to the efficiencies of the street strains 1088 and RABV-Dog and is lower than the efficiencies of the fixed Nishigahara and CVS strains.

### 3.7. Neurovirulence and Neuroinvasiveness of Koma Strain in Mice

To compare the neurovirulence of each of the strains by using a mouse model, we determined their LD_50_ values in mice after i.c. inoculation. The LD_50_ values of the fixed strains Nishigahara and CVS after i.c. inoculation were 20.8 and 48.1 FFU, respectively, while the value of the ERA strain remained undetermined because of its dose-independent lethality in mice (Table 2). The LD_50_ values of the street strains 1088 and RABV-Dog were 10.0 and 15.3 FFU, respectively. These data indicate that neurovirulence of the street strains is comparable to that of the fixed strains. The LD_50_ value of the Koma strain (4.2 FFU) was similar to the values of street 1088 and RABV-Dog strains, indicating that there is no obvious difference in neurovirulence between the Koma strain and these street strains.

Next, to compare the levels of neuroinvasiveness, we determined the LD_50_ values of the strains in mice after i.m. inoculation. The LD_50_ values of the fixed strains Nishigahara, CVS and ERA after i.m. inoculation were 1.5 × 10^5^, 2.4 × 10^5^, and over 1.0 × 10^6^ FFU, respectively (Table 3). The values of the street strains 1088 and RABV-Dog (2.4 × 10^3^ and 3.3 × 10^3^ FFU, respectively) were at least 45-fold lower than the values of the fixed strains, indicating that the street strains are more neuroinvasive than the fixed strains. Importantly, the LD_50_ value of the Koma strain (3.2 × 10^2^ FFU) was also lower than the values of the fixed strains. These findings indicated that the Koma strain has a higher level of neuroinvasiveness than do the fixed strains.

### 3.8. Incubation Period in Koma-Infected Mice

**Figure 7:**
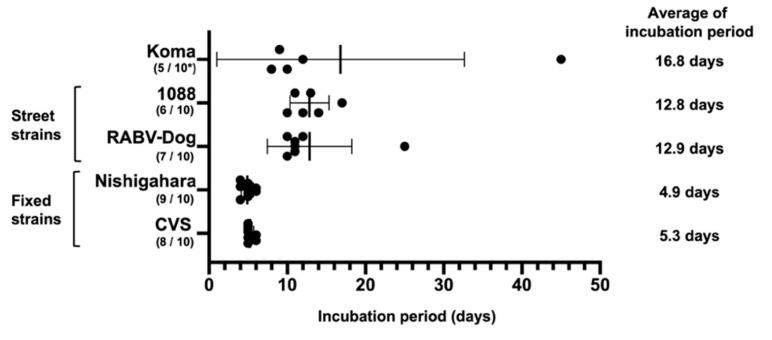
Incubation periods in mice infected with virus strains via the i.m. route. Six-week-old male mice (10 mice/group) were inoculated intramuscularly with 3.0 LD_50_ of each of the strains into the left thigh muscle and were observed daily for 50 days. Each column represents the date of onset of disease in each mouse (mean ± standard deviation). *, number of sick/inoculated mice.

A long and inconstant incubation period is a characteristic of natural rabies infection in humans and animals [1,48,49,50]. To check whether such an incubation period can be observed in mice infected with the Koma strain and street 1088 and RABV-Dog strains, we compared periods from the day of inoculation of the virus strains to the day of onset of disease after i.m. inoculation (Figure 7). In this experiment, six and seven of 10 mice showed clinical signs after i.m. inoculation with street 1088 and RABV-Dog strains, respectively, while nine and eight of 10 mice manifested signs after inoculation with fixed Nishigahara and CVS strains, respectively. Incubation periods in mice infected with street 1088 and RABV-Dog strains ranged from 10 to 17 days (12.8 days on average) and from 10 to 25 days (12.9 days), respectively. The periods in mice infected with fixed Nishigahara and CVS strains ranged from 4 to 6 days (4.9 days) and from 5 to 6 days (5.3 days), respectively. These findings indicated that the incubation periods in mice infected with street 1088 and RABV-Dog strains were longer and more inconstant than the periods in mice infected with fixed Nishigahara and CVS strains. Importantly, in mice infected with the Koma strain (five of 10 inoculated mice), the incubation period was in the range of 8–45 days (16.8 days), indicating that the period was also longer and more inconstant than that in mice infected with the fixed strains. Taken together, the findings demonstrate that the long and inconstant incubation period can be reproduced by using the mouse infection model with the Koma strain, as well as street 1088 and RABV-Dog strains.

### 3.9. Distribution of Virus in the Brains of Mice Infected with Koma Strain via the i.m. Route

**Figure 8:**
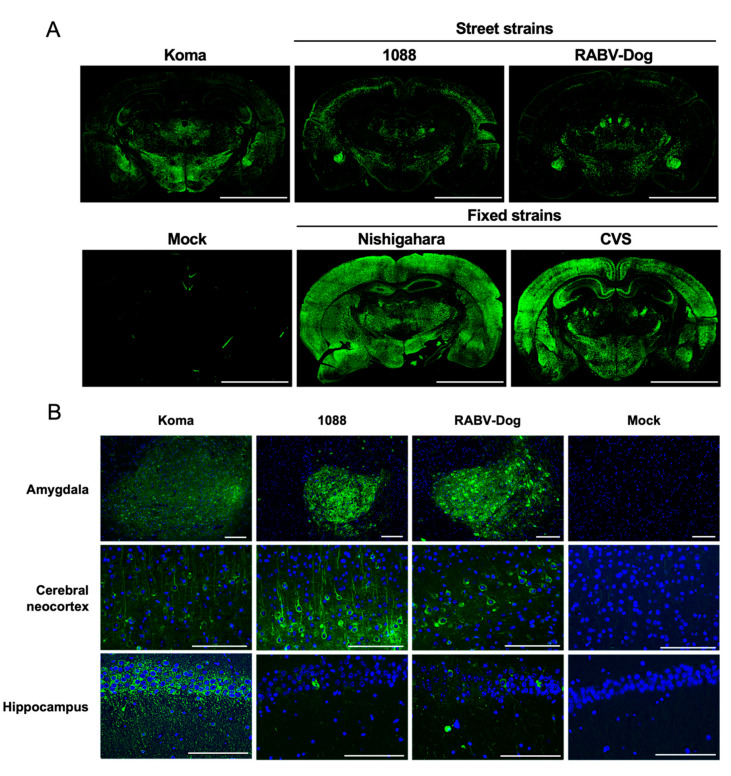
Distribution of viral antigens in the brains of mice infected with the virus strains. The brains were collected from mice infected with each virus strain at the terminal stage of infection. A mock-infected brain was collected at 35 dpi. The brain tissues were stained with an anti-RABV P protein antibody. (**A**) Representative immunofluorescence staining image of RABV P protein (green) in the whole brains of infected mice. The scale bars correspond to 3.5 mm. (**B**) Magnified fields of the amygdala, cerebral neocortex, and hippocampus in mice infected with Koma, 1088, RABV-Dog, or Mock. Nuclei were stained with DAPI (blue). The scale bars correspond to 150 µm.

**Figure A1:**
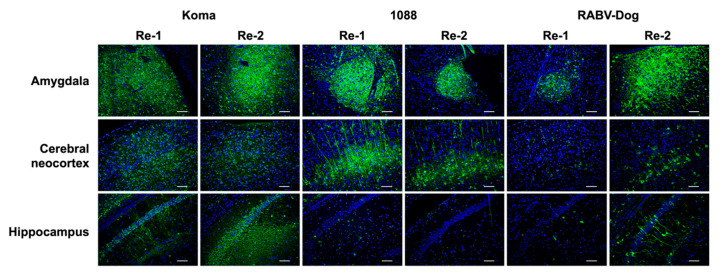
Distribution of viral antigens in the amygdala, cerebral neocortex, and hippocampus in mice infected with the virus strains. The distribution of viral antigens in the brain was re-examined by using each of two brain samples infected with the Koma, 1088, or RABV-Dog strain. Viral protein and nuclei were stained with an anti-RABV P protein antibody (green) and DAPI (blue), respectively. The scale bars correspond to 100 µm. These samples were photographed by using the Biozero fluorescence microscope BZ-8000 series (Keyence).

In order to compare the extents of spread of the virus strains in the brains of mice after i.m. inoculation, we performed immunohistochemical analysis and examined the distributions of virus-infected cells in the brains of mice that were at the terminal stage and were euthanized (Figure 8A). The results indicated that the fixed Nishigahara and CVS strains spread widely through almost all of the brain regions examined (Figure 8A. lower panels). In contrast, the Koma strain and street 1088 and RABV-Dog strains spread less widely than did the fixed strains (Figure 8A. upper panels). Notably, we found that the distribution of infected cells differed among the brains infected with the Koma and the street strains (Figure 8B). More specifically, cells infected with the Koma strain were distributed more intensely in the hippocampus than were cells infected with 1088 and RABV-Dog strains, while cells infected with 1088 strain were more prominent in the cerebral neocortex than were cells infected with the Koma and RABV-Dog strains. Similar results were obtained from separate experiments (Figure A1). Interestingly, cells infected with each of the three strains were prominent in the amygdala, suggesting that infection in this region is important for the pathogenesis of rabies.

## 4. Discussion

In this study, we determined for the first time the complete genome sequence of the RABV strain Koma, which was isolated from a dog in Tokyo, Japan in the 1940s and is considered to be the only currently existing Japanese street strain, and we analyzed the genome sequence to understand the genetic properties of this strain including its phylogenetic origin. The results of genome-wide phylogenetic analysis indicate that the Koma strain is genetically closely related to RABV strains of the Arctic-related clade, especially to RV303 strain isolated from a raccoon dog in Primorsky Krai, the Far East region of Russia (Figure 2), being consistent with the results of analysis based on partial and whole nucleotide sequences of the N gene [33,38]. More importantly, the complete genome sequences of the Koma and RV303 strains have as high as 98.2% homology at the nucleotide level (Table 1), strongly suggesting that the two strains were derived from a common ancestor, despite a geographical barrier between Tokyo and Primorsky Krai (i.e., the Sea of Japan). A previous phylogenetic study based on the N gene sequences indicated the possibility that an ancestor virus of the Koma strain was introduced from the region of Russia to Japan through active economic and military transports, lasting until the end of the World War II [33]. Our findings described above are consistent with and further support this possibility.

A comparison of the amino acid sequences of all five viral proteins demonstrated that, surprisingly, there are only 17 amino acid differences between the Koma strain and the street RV303 strain (Figure 3), despite the fact that the Koma strain was isolated from a dog before being serially passaged in rodents [32], while RV303 strain was isolated from a raccoon dog [38]. Although the impacts of those differences on the structures and functions of the respective proteins and also on viral phenotypes remain unknown, we speculate that some of those are potentially associated with adaptation to their host species (i.e., dog/rodent and raccoon dog, respectively). Notably, six of the 17 amino acid differences are accumulated in the P protein (Figure 3), which is known to be a viral interferon antagonist [6,7,9,45,51,52]. The antagonist activity might be modified by those mutations to achieve adaptation to the host species. 

The genome-wide analysis revealed that the Koma strain has genetic features that closely resemble those of the street RV303 strain (Table 1, Figure 3) and also that the Koma strain has two potential N-glycosylation sites on the G protein as most of the street strains do (Figure 4), leading to the hypothesis that the Koma strain retains biological phenotypes similar to those of street strains. To test this hypothesis, we examined and compared in vitro and in vivo phenotypes of the Koma strain comprehensively with those of representative street strains (1088, RABV-Dog), as well as those of fixed strains (Nishigahara, CVS, ERA). 

The results of our phenotypic examinations indicated that, consistent with the above-stated hypothesis, the phenotypes of the Koma strain are more similar to those of these street strains than those of the fixed strains (summarized in Table 4). In short, as in the case of the street strains, the Koma strain is less neurotropic and more neuroinvasive than the fixed strains. These findings led to the conclusion that the Koma strain has not been “fixed” yet, although this strain has undergone serial passages in animal brains as most of fixed strains have. Lépine [53] previously reported that it usually requires approximately 80 passages in animal brains for typical street strains to become “fixed”. Since the Koma strain used in this study underwent only 24 passages in mouse brains after 19 brain-to-periphery passages in guinea pigs, further brain-to-brain passages in animal brains would be needed to fix this strain.

In this study, we characterized phenotypes of the RABV strains in vitro, and found that propagation and cell-to-cell spread of the street 1088 and RABV-Dog strains were less efficient in mouse neuroblastoma NA cells than were those of the fixed Nishigahara and CVS strains (Figure 5 and Figure 6). This difference is likely to be related to the in vivo findings that infection with these street strains spread less efficiently in the mouse brains than did infection with the fixed strains (Figure 8). These findings indicate that the difference in neurotropism can be a standard for phenotypically dividing into street and fixed strains. However, our data demonstrate that, surprisingly, this difference does not drastically affect neurovirulence of the street and fixed strains: there was no obvious difference between the LD_50_ values of the street and fixed strains in mice after i.c. inoculation (Table 2). Similar results were previously obtained by phenotypic characterizations of the street 1088 strain and its mutants, which had acquired higher neurotropism by serial passages in NA cells [23,54,55]. In contrast, the results of in vivo characterization highlight an obvious difference in the neuroinvasiveness between the street and fixed strains: the LD_50_ values of the street 1088 and RABV-Dog strains in mice after i.m. inoculation were clearly lower than those of the fixed Nishigahara and CVS strains (Table 3). Although further studies are needed to determine whether and how the difference in neurotropism affects the neuroinvasiveness of respective strains, it seems reasonable to assume that fixed strains have lost a certain mechanism to evade host immunity in the peripheral tissue through serial passages in animal brains. 

A long and inconsistent incubation period is one of characteristics of natural rabies infection [48,50]. In this study, we observed that mice inoculated with the Koma strain and the street 1088 and RABV-Dog strains showed clinical signs after long and inconstant incubation periods (Figure 7), suggesting that the i.m. inoculation of mice with these three strains provides a good model reflecting natural infection. The results of a previous study obtained by using a street strain suggested that the virus stays at or near the site of inoculation during most of the long incubation period [56]. Consistent with this finding, Charlton et al. [49] previously demonstrated, by inoculating striped skunks with a street strain, that viral genomic RNAs and viral antigen were detected in the inoculated muscle before the inoculated skunks showed clinical signs. Although these findings suggest that infection of muscle cells contributes to the long and inconstant incubation period, little is known about the infection dynamics and mechanisms of RABV in peripheral tissue. We believe that the Koma strain, as well as the street 1088 and RABV-Dog strains will be useful tools for elucidating the mechanisms underlying the long and inconstant incubation period, which is usually observed in rabies cases in humans and animals.

In this study, we examined the distribution of infected cells in mouse brains after i.m. inoculation with the Koma strain and the street 1088 and RABV-Dog strains and we found that cells infected with 1088 strain were distributed more intensively in the cerebral neocortex than were cells infected with the Koma and RABV-Dog strains, whereas infection with the Koma strain was more prominent in the hippocampus than was infection with the 1088 and RABV-Dog strains (Figure 8). These differences indicate the possibilities that the respective strains spread differently in the brain tissue and, alternatively, that these strains spread to the CNS via different routes in the neuron network from a peripheral inoculation site. Considering that all of these three strains have similar levels of pathogenicity, equally causing lethal infection in mice after i.m. inoculation (Table 3), we believe that these differences are associated not with the intrinsic mechanisms of rabies pathogenesis, but only with minor variations in the disease phenotype. To elucidate the intrinsic pathogenic mechanisms, it will be important to specify the brain areas that are commonly affected by infection with any street strains. Interestingly, our findings indicate that infections with all of the three strains were commonly concentrated in the amygdala area (Figure 8B), which plays an important role in emotional responses including fear, anxiety and aggression [57]. We therefore hypothesized that infection in this area may be involved in aggressive behaviors that are frequently observed in humans and animals infected with RABV [58,59]. In order to obtain clues for elucidation of the pathogenic mechanisms of rabies, in the near future, we will exhaustively compare distributions of cells infected with the Koma, 1088 and RABV-Dog strains in mouse brains, by using a 3D imaging technique that was reported recently [24,60]. 

In conclusion, the findings obtained in this study indicate that the Koma strain belongs to the Arctic-related clade and retains genetic and phenotypic properties similar to those of street strains. Although further studies will be needed to check whether this strain is phenotypically similar to the Arctic-related strains currently circulating in nature, of which biological phenotypes including their pathogenicity are largely unknown [61], the findings in this study strongly suggest that the Koma strain is a useful tool for studying the pathogenesis of rabies. Importantly, our data also demonstrated phenotypic differences in vivo of the respective RABV strains including the Koma strain, especially in the spread of infection in the brain. This clearly indicates the necessity for comparative studies of genetically diverse street strains to understand the intrinsic mechanisms of rabies pathogenesis. More specifically, although several RABV strains within the Cosmopolitan and Arctic-related clades were compared in this study, the comparison needs to be extended to viral strains of other clades in the future. It is also important to include primary viral isolates directly obtained from naturally infected animals for future comparative studies. Such studies will provide fundamental information that is necessary for the development of a therapeutic approach and a novel prophylaxis method for rabies.

## Figures and Tables

**Table 1:** Homology of each viral gene between the Koma and RV303 at nucleotide and amino acid levels.

	Total	N Gene	P Gene	M Gene	G Gene	L Gene
Nucleotide	98.2%	98.5%	97.2%	97.7%	98.1%	98.3%
Amino acid	99.5% ^1^	99.8%	98.0%	99.0%	99.4%	99.8%

^1^ Average of amino acid homologies of all five viral proteins.

**Table 2:** Pathogenicity of each of the virus strains in mice after i.c. inoculation.

Dose	Koma	1088	RABV-Dog	Nishigahara	CVS	ERA
**1.0 × 10^3^ FFU**	NT	NT	NT	100% (5/5) ^1^	100% (5/5)	60% (3/5)
**1.0 × 10^2^ FFU**	100% (10/10)	100% (10/10)	70% (7/10)	80% (4/5)	60% (3/5)	100% (5/5)
**1.0 × 10^1^ FFU**	80% (8/10)	50% (5/10)	50% (5/10)	40% (2/5)	20% (1/5)	20% (1/5)
**1.0 × 10^0^ FFU**	0% (0/10)	0% (0/10)	10% (1/10)	0% (0/5)	0% (0/5)	0% (0/5)
**LD_50_ (FFU)**	4.2	10.0	15.3	20.8	48.1	ND

^1^ Mortality rate (No. dead/inoculated), NT: not tested, ND: not determined.

**Table 3:** Pathogenicity of each of the virus strains in mice after i.m. inoculation ^1^**.**

Dose	Koma	1088	RABV-Dog	Nishigahara	CVS	ERA
**1.0 × 10^6^ FFU**	NT	NT	NT	100% (5/5) ^2^	100% (5/5)	40% (2/5)
**1.0 × 10^5^ FFU**	NT	NT	NT	40% (2/5)	20% (1/5)	20% (1/5)
**1.0 × 10^4^ FFU**	NT	100% (10/10)	60% (6/10)	0% (0/5)	0% (0/5)	0% (0/5)
**1.0 × 10^3^ FFU**	80% (8/10)	20% (2/10)	30% (3/10)	NT	NT	NT
**1.0 × 10^2^ FFU**	20% (2/10)	0% (0/10)	10% (1/10)	NT	NT	NT
**LD_50_ (FFU)**	3.2 × 10^2^	2.4 × 10^3^	3.3 × 10^3^	1.5 × 10^5^	2.4 × 10^5^	>1.0 × 10^6^

^1^ Each of the virus strains was inoculated into the left thigh muscle of six-week-old male mice. ^2^ Mortality rate (No. dead/inoculated), NT: not tested.

**Table 4:** Differences between the phenotypic properties of street viruses and fixed viruses.

	Fixed Viruses (FVs) vs. Street Viruses (SVs) vs. Koma Strain
**Growth in neuroblastoma cells**	FVs > SVs = Koma
**Cell-to-cell spread in neuroblastoma cells**	FVs ^1^ > SVs = Koma
**Neurovirulence in mice (i.c. route)**	FVs = SVs = Koma
**Neuroinvasiveness in mice (i.m. route)**	FVs < SVs = Koma
**Incubation period in mice (i.m. route)**	FVs (Constant) < SVs = Koma (Inconstant)
**Virus spread in the mouse brain**	FVs > SVs ^2^ = Koma ^2^

^1^ Except for ERA strain, ^2^ Differently distributed in the mouse brains.

## Supplementary Materials

The following are available online at https://www.mdpi.com/1999-4915/12/9/914/s1, Table S1: Primers used in this study, Table S2: Rabies virus strains used for phylogenetic analysis.
